# Antimicrobial resistance and biofilm formation in rarely reported *Salmonella enterica* serovars from patients presenting with gastroenteritis in Nairobi, Kenya

**DOI:** 10.3389/fmicb.2025.1628784

**Published:** 2025-06-25

**Authors:** Peter Muturi, Cecilia Mbae, Evans Kibet, Peter Njoroge, Susan M. Kavai, Darius Ideke, Jessicah Jepchirchir, John S. Gunn, Samuel Kariuki

**Affiliations:** ^1^Centre for Microbiology Research, Kenya Medical Research Institute, Nairobi, Kenya; ^2^Center for Microbial Pathogenesis, Abigail Wexner Research Institute at Nationwide Children's Hospital, Columbus, OH, United States; ^3^Infectious Diseases Institute, The Ohio State University, Columbus, OH, United States; ^4^Eastern Africa Office, Drugs for Neglected Diseases Initiative, Nairobi, Kenya

**Keywords:** non-typhoidal, *Salmonella*, AMR genes, biofilms, Kenya

## Abstract

Non-typhoidal *Salmonella* infections are a significant global public health concern, causing approximately 150 million illnesses and 60,000 deaths annually, with majority of the cases occurring in low- and middle-income countries. In this study, we used whole genome sequencing to identify and characterize uncommon non-typhoidal *Salmonella* serovars isolated from patients presenting with gastrointestinal symptoms in the Mukuru area of Nairobi, Kenya. Sixteen less common NTS serovars (excluding *Salmonella* Typhimurium and *S*. Enteritidis) were identified from 25 patients, with 1 isolate from blood and 24 from stool samples. The most common serovar was *S*. Newport, isolated from 6 of the 25 patients, followed by *S*. Breda (2 patients), *S*. Eastbourne (2 patients), *S*. Orion (2 patients) and 12 other serovars, each isolated from a single individual. These serovars displayed diverse antigenic profiles, grouped into 9 distinct serogroups. Antimicrobial resistance profiles and *in vitro* biofilm formation of the isolates were also assessed. Antimicrobial resistance was detected in three *S*. Newport strains: two sequence type 31 (ST31) isolates carried the *bla*_*TEM*−1_ and *tet(A)* resistance genes, while one ST166 isolate carried *bla*_*TEM*−1_, *tet(A), aph(6)-Id*, and *sul2*. Biofilm formation varied among the serovars and was enhanced by cholesterol while inhibited by bile. Strong biofilm formation was observed in *S*. Breda, *S*. Hann, and *S*. Eastbourne, whereas *S*. Chicago and *S*. Kentucky formed weak biofilms. This study highlights the diversity of NTS serovars circulating in Nairobi and emphasizes on the importance of localized studies in addressing regional variations in NTS epidemiology. To effectively mitigate the burden of NTS infections and curb the spread of AMR, sustained genomic surveillance, the development of advanced diagnostic tools for emerging *S. enterica* infections, and the implementation of integrated public health interventions are essential.

## 1 Introduction

Non-typhoidal *Salmonella* (NTS) infections, caused by *Salmonella enterica* strains other than *S*. Typhi and *S*. Paratyphi, remains a significant global public health challenge. NTS are estimated to cause approximately 150 million illnesses and 60,000 deaths globally each year (Sima et al., 2024). The majority of these cases occur in low- and middle-income countries, particularly in sub-Saharan Africa (sSA), where poor sanitation and limited access to clean drinking water facilitate the rapid spread of infection, leading to high morbidity and mortality rates (Gilchrist and MacLennan, 2019). These outcomes are often linked to therapeutic failures, which have become more prevalent in recent years due to the increasing antimicrobial resistance in *Salmonella* strains (Tyson et al., 2021).

The genus *Salmonella*, belonging to the family *Enterobacteriaceae*, comprises two species: *Salmonella enterica* and *Salmonella bongori*. The latter species is predominantly associated with cold-blooded animals and is considered a rare opportunistic pathogen in humans (Gal-Mor, 2018). In contrast, *S. enterica* is further classified into six subspecies (I–VI) and over 2,500 serotypes or serovars, based on biochemical, antigenic, and serological properties (Haeusler and Curtis, 2013). While *Salmonella* Typhi and *Salmonella* Paratyphi are human-restricted pathogens, other *S. enterica* serotypes have broad host ranges, infecting various domestic and wild animals while also causing disease in humans (Gal-Mor, 2018).

The geographical distribution of NTS serovars is shaped by various factors, including climate, agricultural practices, food supply chains, and local epidemiological dynamics. Notable NTS serovars with significant epidemiological impact include *S*. Typhimurium, *S*. Enteritidis, *S*. Heidelberg, and *S*. Newport (Ferrari et al., 2019; Shariat et al., 2013). In sSA, invasive non-typhoidal salmonelloses (iNTS) are predominantly caused by two serovars: *S*. Typhimurium and *S*. Enteritidis (Kariuki et al., 2019; Kasumba et al., 2023; Okoro et al., 2012). The global incidence of iNTS disease is underreported due to limited diagnostic capacity, as accurate identification requires well-equipped microbiology laboratories and skilled technicians, both of which are often unavailable in resource-limited settings (Balasubramanian et al., 2019).

*Salmonella* is primarily transmitted via the fecal-oral route, often due to inadequate hand hygiene (Centers for Disease Control Prevention., 2024). NTS typically colonize the gastrointestinal tract, causing self-limiting diarrhea in humans. However, certain strains can invade the bloodstream, leading to iNTS, a severe systemic infection that poses a significant risk to immunocompromised individuals and young children (Caro-Castro et al., 2024; Cuypers et al., 2018).

*Salmonella* spp. can form biofilms on both biotic and abiotic surfaces, a well-documented form of bacterial growth (Steenackers et al., 2012; Stepanović et al., 2004). They exhibit a cyclic lifecycle, alternating between host colonization and environmental persistence (Winfield and Groisman, 2003). Biofilm formation is clinically significant, with approximately 80% of chronic bacterial infections linked to this mode of growth (Hall-Stoodley and Stoodley, 2009; Steenackers et al., 2012). It enhances bacterial survival by increasing resistance to antimicrobial agents and evading immune defenses, contributing to chronic and device-related infections (Cangui-Panchi et al., 2023; González et al., 2018; Mirghani et al., 2022).

*S*. Typhimurium has been shown to form robust biofilms in mice fed a lithogenic diet, leading to asymptomatic infections. In these models, a strong association was observed between bacterial shedding and the presence of bacteria in the cecum and large intestines (Crawford et al., 2010; Cruz-Cruz et al., 2025). In contrast, *S*. Typhi, the causative agent of typhoid fever, forms strong biofilms *in vitro* under conditions that simulate the human gallbladder environment, particularly in the presence of cholesterol and bile. Although NTS serovars have not been shown to colonize the human gallbladder, they are likely to encounter bile in the small intestines following its release from the gallbladder after meals, where it plays a critical role in lipid emulsification (Ørntoft et al., 2021). Few studies have investigated the biofilm-forming ability of the less common NTS serovars. However, variations in biofilm formation and virulence among iNTS strains have been documented (Vasicek and Gunn, 2023).

Information on the full range of NTS serovars isolated from patients with gastroenteritis in sSA remains limited, largely due to the unavailability of antisera needed to identify less commonly isolated serovars in the region. In this study, we utilized whole genome sequencing to identify and characterize NTS serovars, excluding *S*. Typhimurium and *S*. Enteritidis, isolated from human and blood samples collected in Nairobi, Kenya. Additionally, we evaluated the antimicrobial susceptibility and biofilm-forming ability of the identified serovars.

## 2 Materials and methods

### 2.1 Study participants and sample collection

Patients aged 12 years and older presenting with symptoms of salmonellosis, such as diarrhea, abdominal pain, or fever of unknown origin, were enrolled in the study. Participants were recruited from four primary health centers in the Mukuru kwa Njenga and Mukuru kwa Ruben informal settlements, as well as from Mama Lucy Kibaki Hospital, a Level-5 county referral facility in Nairobi, Kenya. The study was conducted between December 2020 and June 2023. Blood and stool samples were collected from participants, following their consent, for *Salmonella* culture and antimicrobial susceptibility testing (AST). Metadata, including the nearest landmark, were recorded and subsequently used to capture Global Positioning System (GPS) coordinates using the Epicollect5 data collection tool on a study mobile phone. To ensure optimal pathogen recovery, a sterile swab was used to aliquot a portion of each stool specimen into Cary-Blair transport medium. The specimens were then sealed in zip-lock bags and placed in a cooler box maintained at 2–8°C for transport to the laboratory at KEMRI.

Blood samples were collected for BACTEC *Salmonella* identification following standard procedures. The venipuncture site was disinfected using 70% alcohol swabs and allowed to stand for 30 s to minimize contamination. Using a sterile syringe, 8–10 mL of blood was drawn from the patients. The blood was immediately inoculated aseptically into BACTEC aerobic culture bottles and gently mixed to prevent clotting. Labeled samples were promptly transported to the laboratory at CMR, KEMRI, ensuring that room temperature was maintained.

### 2.2 Stool and blood culture

The aliquoted stool sample on the cotton swab was inoculated into Selenite Feacal (SF) broth, an enrichment medium, and incubated aerobically at 37°C for 18–24 h. Following incubation, a loopful of the SF broth was streaked onto Xylose Lysine Deoxycholate (XLD) agar and incubated aerobically at 37°C for another 18–24 h. BACTEC vials containing blood samples were placed in the BACTEC blood culture system for incubation and detection of positive blood cultures. All positive blood cultures were subsequently streaked onto blood agar (BA) and XLD agar, followed by aerobic incubation at 37°C for 18–24 h. Non-lactose fermenting colonies, identified by their distinct brick-red appearance or brick-red colonies with black centers on XLD agar, were sub-cultured onto Tryptone Soya Agar (TSA), a non-selective medium. The colonies were characterized phenotypically using standard biochemical tests, including Triple Sugar Iron (TSI) agar, urea hydrolysis, and motility tests, and further confirmed using the Analytical Profile Index (API) system (Montalieu Vercieu, France).

*Salmonella* isolates were confirmed using the White-Kauffmann-Le Minor scheme (Grimont and Weil, 2007; Guibourdenche et al., 2010) with *Salmonella* polyvalent O antisera. Due to the unavailability of monovalent antisera in our laboratory for identifying less common *Salmonella* serovars, isolates that did not exhibit the characteristic antigenic profiles of *S*. Typhi (O:9, Vi:d:-), *S*. Typhimurium (4:i:1,2), or *S*. Enteritidis (9:g,m:-) were subjected to genomic DNA extraction, followed by whole genome sequencing for serovar identification and detection of antimicrobial resistance genes.

### 2.3 Antimicrobial susceptibility testing

Antimicrobial susceptibility testing was performed using the disk diffusion method (Reller et al., 2009) for a range of antimicrobials commonly used to treat *Salmonella* infections, including ampicillin (10 μg), tetracycline (30 μg), co-trimoxazole (25 μg), chloramphenicol (30 μg), amoxicillin–clavulanate (20/10 μg), cefpodoxime (30 μg), ceftazidime (30 μg), ceftriaxone (30 μg), cefotaxime (30 μg), azithromycin (15 mg), ciprofloxacin (5 μg), nalidixic acid (10 μg), kanamycin (30 mg), and gentamicin (10 mg). The diameter of the zone of inhibition was measured after 18–24 h using a Vernier caliper, and results were interpreted in accordance with the Clinical and Laboratory Standards Institute (CLSI) guidelines for *Salmonella* (Performance Standards for Antimicrobial Susceptibility Testing (33rd ed.) (2023) (Clinical Laboratory Standards Institute., 2023).

### 2.4 DNA extraction and whole genome sequencing

DNA was extracted from *Salmonella* isolates using the GenElute™ Bacterial Genomic DNA Kit (Sigma-Aldrich, Missouri, USA) for whole genome sequencing (WGS), following the manufacturer's instructions. Library preparation for WGS was performed by SeqCoast Genomics (Portsmouth, New Hampshire, USA) using the Illumina DNA Prep Tagmentation Kit with unique dual indexes. Sequencing was conducted on the Illumina NextSeq 2000 platform with a 300-cycle flow cell kit, generating 2 × 150-bp paired-end reads, as previously described (Grant et al., 2023). A PhiX control (1%−2%) was included in the sequencing run to optimize base calling. Demultiplexing, read trimming, and run analytics were carried out using DRAGEN v3.10.12, an onboard analysis software of the NextSeq 2000.

### 2.5 Genome assembly and annotation

Raw reads were quality-trimmed using Trimmomatic (version 0.39) (Bolger et al., 2014), followed by error correction using SPAdes (version 3.13.1) (Bankevich et al., 2012). Assembly into contigs was performed using SPAdes, wrapped in Unicycler (version 0.4.4) (Wick et al., 2017), with read mapping conducted using Bowtie2 (Langmead and Salzberg, 2012) and alignment processing utilizing SAMtools (Li et al., 2009). Assembly polishing was carried out with Pilon (version 1.24) (Walker et al., 2014), also wrapped in Unicycler. Gene prediction and functional annotation were performed using BAKTA (version 1.5.1) (Schwengers et al., 2021). The annotation pipeline was as follows: prediction of protein-coding genes using Prodigal, tRNA identification using tRNAscan-SE (Chan and Lowe, 2019), tRNA and tmRNA identification using Aragorn (Laslett and Canback, 2004), prediction of rRNA sequences using Infernal and the Rfam database (Nawrocki and Eddy, 2013), CRISPR prediction using PILER-CR (Edgar, 2007), antimicrobial resistance gene identification using AMRFinderPlus (Feldgarden et al., 2021), prediction of signal peptides using DeepSig (Savojardo et al., 2018), prediction of transposases using ISFinder (Siguier et al., 2006), and computation of codon usage biases for each amino acid using the codonUsage.py script (Garber, 2024) available at https://github.com/Arkadiy-Garber/BagOfTricks.

### 2.6 Identification of *Salmonella enterica* serovars

Enterobase *Salmonella* database, a web-based platform for genome analysis accessible at https://enterobase.warwick.ac.uk/species/index/senterica, was used for the identification of NTS serovars (Zhou et al., 2020). The platform utilizes WGS data to assign *Salmonella* strains to specific serovars through its integration with tools like SISTR (*Salmonella in Silico* Typing Resource) (Yoshida et al., 2016) and Multi-Locus Sequence Typing (Achtman et al., 2012). Enterobase combines the functionalities of both SISTR1 and SeqSero2 for *in silico* serotyping of *Salmonella* genomes (Zhou et al., 2020). WGS data in FASTA format were uploaded to the database for analysis. Results obtained for each strain included the serovar name, sequence type and predicted antigenic profile. Enterobase provided predictions for the expression of the O antigen, as well as the flagellar antigens H1 and H2, based on the analysis of the O antigen gene cluster and the *fliC* and *fljB* genes.

### 2.7 Phylogenetic tree

The Enterobase SNP (Single Nucleotide Polymorphism) phylogenetic tree web-based tool (Alikhan et al., 2018) was used to construct a phylogenetic tree for the *Salmonella* isolates based on whole genome sequences. This analysis aimed to investigate the genetic relationships and evolutionary patterns of the isolated *Salmonella* strains, alongside several additional strains from different regions with available whole genome sequences from the NCBI public database. *Salmonella bongori* (GenBank Accession: SAMN43895974) was included as the outgroup. The resulting phylogenetic tree was downloaded in Nerwick format and uploaded to Microreact (https://microreact.org/) for further visualization. Associated metadata was exported in CSV format to Microreact, and a Newick-format phylogenetic tree was generated, reflecting branch lengths that correspond to SNP/genetic distances.

### 2.8 Screening of antimicrobial resistance genes

Acquired antimicrobial resistance genes and known resistance-associated point mutations in the *Salmonella*-assembled nucleotide sequences were identified using NCBI's Antimicrobial Resistance Gene Finder Plus (AMRFinderPlus, v3.12.8) (Feldgarden et al., 2021), as outlined in the tool's documentation (https://github.com/ncbi/amr/wiki).

### 2.9 Plasmid identification

To identify plasmids carrying acquired antimicrobial resistance (AMR) genes in the genomic DNA of isolated *Salmonella* strains, the PLASMe plasmid detection tool (v1.1) was used (https://github.com/HubertTang/PLASMe). The tool employs an alignment component to detect closely related plasmids, while order-specific Transformer models predict more diverged plasmids (Tang et al., 2023). Assembled *Salmonella* genomes were used for this analysis.

### 2.10 *In vitro* biofilm growth

*Salmonella* biofilms were grown on non-treated polystyrene 96-well plates (Corning, Kennebunkport, ME) as previously described (González et al., 2018). The biofilm formation was evaluated under four conditions: in the absence of both cholesterol and bile, in the presence of cholesterol but without bile, in the presence of bile but without cholesterol, and in the presence of both cholesterol and bile. Cholesterol was incorporated by precoating the wells with cholesterol solution (prepared by mixing 5 mg/mL cholesterol and 1:1 isopropanol:ethanol) which was then air-dried overnight (Crawford et al., 2008). A single colony of non-typhoidal *Salmonella* strain, grown on XLD agar, was inoculated into 3 mL of TSB and incubated overnight at 37°C with gentle rolling. The OD_600_ of the overnight culture was measured using a NanoDrop spectrophotometer (TSB served as the blank) and normalized to OD_600_ = 0.8 in 1:20 TSB. The normalized culture was then further diluted 1:10 in 1:20 TSB or 1:20 TSB containing 2.5% human bile, and 100 μL of the resulting suspension was added to each well of the microtiter plate (three wells per isolate). The plates were incubated at 25°C on a Fisherbrand™ nutating mixer (Thermo Fisher Scientific, Hampton, NH) set to 24 rpm for 24 h. After incubation, the plates were emptied and washed twice in a bucket of reverse osmosis (RO) water before heat-fixing at 60°C for 1 h. Biofilms were then stained with crystal violet solution (33.33% crystal violet, 60% 1xPBS, 33.33% methanol and 3.33% isopropanol), and bound crystal violet was eluted using 33% acetic acid. The OD_570_ of the eluted solution was measured to quantify biofilm formation. Each strain was analyzed in triplicate, and the entire process was repeated at least three times for each of the four conditions.

GraphPad Prism 9.5 was used to analyze the biofilm formation data. One-way analysis of variance (ANOVA) was performed to assess the significance of differences in biofilm formation across the four different *in vitro* conditions. To assess differences between specific pairs of conditions, Student's *t*-test was applied. The following comparisons were made: absence of both cholesterol and bile vs. presence of cholesterol, and presence of bile vs. presence of both bile and cholesterol. A *p*-value of less than 0.05 (*p* < 0.05) was considered statistically significant.

### 2.11 Ethical statement

#### 2.11.1 Ethical approval

This study was conducted in accordance with the ethical standards and guidelines of Scientific and Research Unit of Kenya Medical Research Institute (KEMRI).

#### 2.11.2 Informed consent

The participants provided their written informed consent to participate in this study.

#### 2.11.3 Laboratory protocols

The biofilm samples were handled following strict laboratory protocols to ensure participant safety and environmental compliance.

## 3 Results

### 3.1 Non-typhoidal *Salmonella* patients

**Figure 1:**
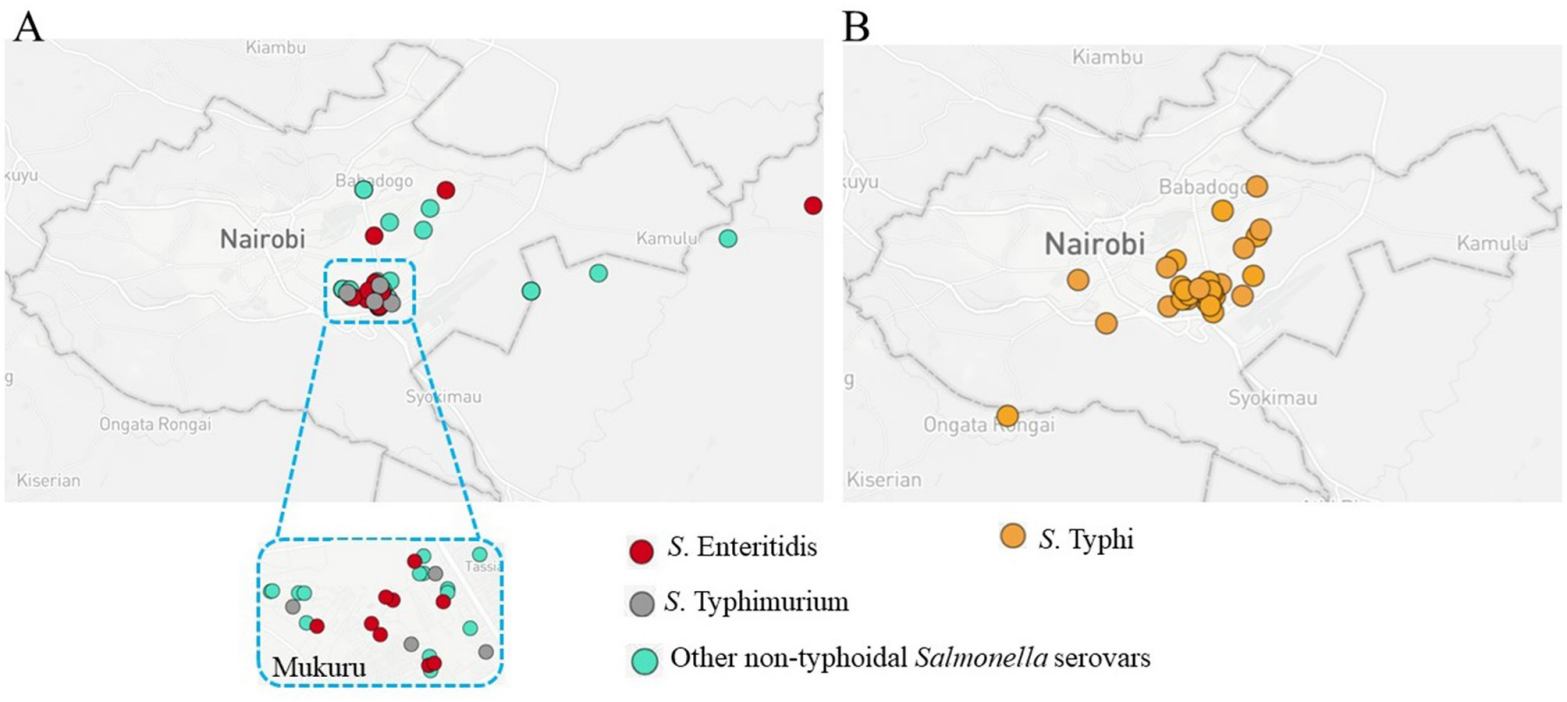
Spatial distribution of *Salmonella* infections in Nairobi, highlighting key hotspots. **(A)** Map of Nairobi showing the distribution of non-typhoidal *Salmonella* (NTS) serovars. Red circles represent *S*. Enteritidis, gray circles represent *S*. Typhimurium, and green circles represent other NTS serovars. A zoomed-in inset highlights the Mukuru slums, an area with a high case density. **(B)** Distribution of culture-positive typhoid fever cases, represented by orange circles (Muturi et al., 2024a).

Between December 2020 and June 2024, a total of 2,634 patients aged ≥12 years old were recruited for the study to identify *Salmonella* infections and detect asymptomatic carriage of *S*. Typhi (Muturi et al., 2024a). Among these, 25 (0.9% positivity rate) had NTS species other than *S*. Typhimurium and *S*. Enteritidis isolated from their stool or blood samples. A concurrent study by our group, conducted in the same setting and period, reported a 1.5% isolation rate of *S*. Enteritidis and *S*. Typhimurium among children under 5 years of age (Kering et al., 2024). Of the 25 patients, 8 were female, and 17 were male. The age distribution of the patients was as follows: 5 were under 20 years of age, 9 were aged 20–29 years, another 9 were in their 30 s, and 2 were over 40 years old. The patients exhibited a range of gastrointestinal and systemic symptoms, including abdominal pain, diarrhea, vomiting, headache, fever, abdominal distention, and cough (Supplementary Table 1). Among the isolates, 24 were recovered from stool samples, while only one was isolated from a blood sample. During the same period, *S*. Typhi infections were confirmed in 1.5% of patients aged ≥12 years old presenting with typical symptoms of typhoid fever (Muturi et al., 2024a). As illustrated in Figure 1, areas identified as hotspots for NTS infections coincided with hotspots for typhoid fever.

### 3.2 Non-typhoidal *Salmonella* serovars

**Figure 2:**
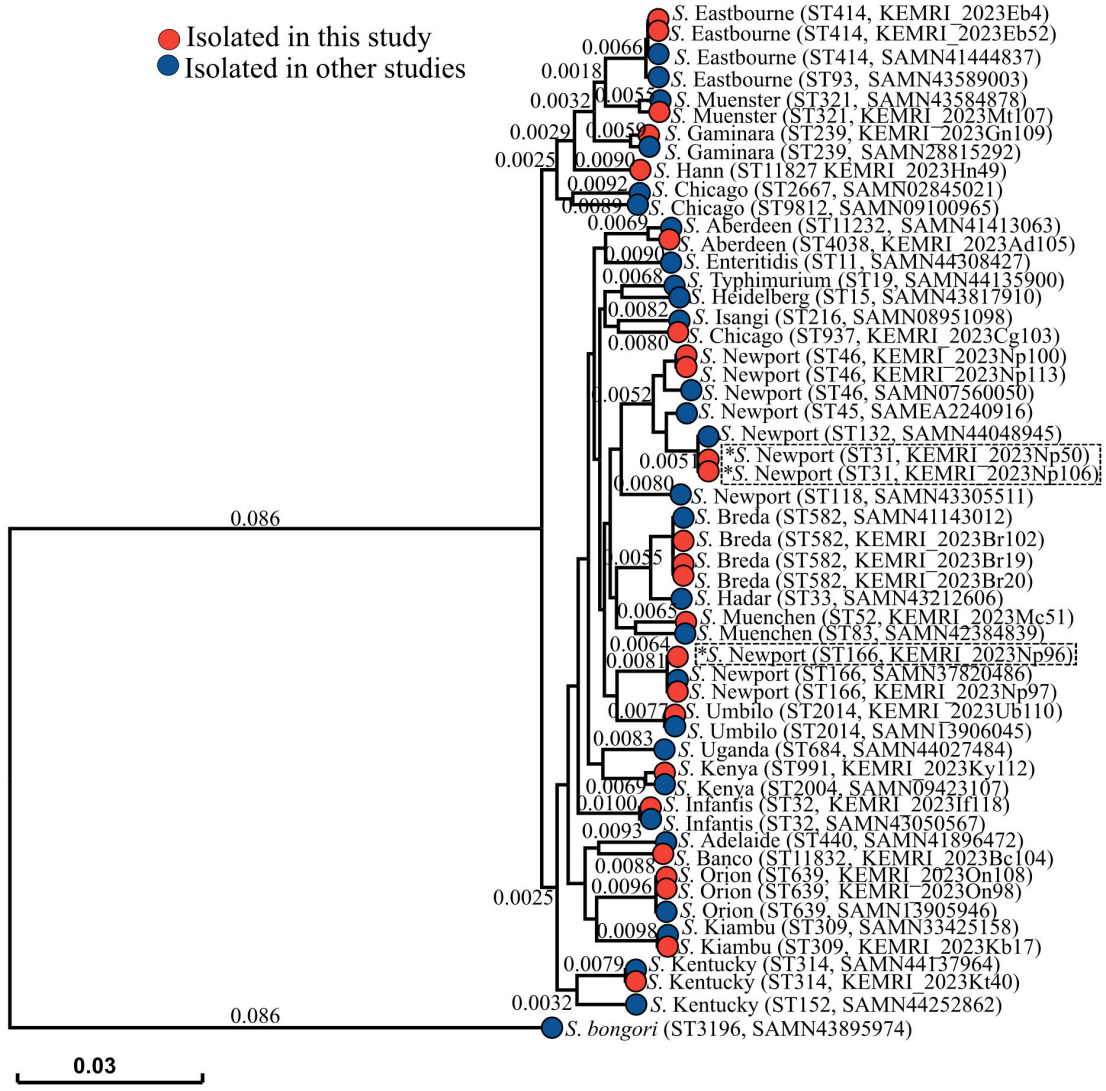
Phylogenetic tree illustrating the evolutionary relationships between NTS strains from this study (represented by red branch tips) and strains from other studies (represented by blue branch tips), including the outgroup *S*. bongori. Sequence types and strain numbers (for isolates from this study) or GenBank accession numbers (for reference strains) are provided in brackets. The scale bar represents the branch length, corresponding to the SNP/genetic distance, which indicates the evolutionary distance between isolates. Isolates carrying AMR genes are marked with an asterisk. An interactive version of the tree can be visualized here https://microreact.org/project/eRMXaPka2DeXRD4Wxm4pqg-nts-serovars-nairobi-2023.

A total of 16 NTS serovars, rarely reported in this region, were isolated from samples collected from 25 patients. *S*. Newport was the most prevalent, from six participants (4 male and 2 Female), followed by *S*. Breda (3 patients; 2 female and 1 male), *S*. Eastbourne (2 cases: both male), and *S*. Orion (2 patients: both male) (Table 1). Each of the remaining 12 participants had a distinct *Salmonella* serovar isolated from their samples, representing 12 additional serovars (Figure 2). Notably, *S*. Newport isolates were classified into three sequence types: ST46, ST31, and ST166. These serovars displayed different antigenic profiles and were classified into 9 distinct serogroups (Table 2).

**Table 1:** Serovars of isolated non-typhoidal *Salmonella* isolates, patient demographics, sample type and collection dates.

**Strain**	***Salmonella* serovar**	**Sequence type**	**Patient's age (years)**	**Patient's gender**	**Sample type**	**Month and year of collection**
KEMRI_2023Np100	*S*. Newport	46	21	Male	Stool	February, 2022
KEMRI_2023Np113	*S*. Newport	46	36	Male	Stool	March, 2023
KEMRI_2023Np50^*^	*S*. Newport	31	53	Female	Stool	April, 2022
KEMRI_2023Np106^*^	*S*. Newport	31	33	Male	Stool	November, 2021
KEMRI_2023Np96^*^	*S*. Newport	166	35	Female	Stool	May, 2022
KEMRI_2023Np97	*S*. Newport	166	14	Male	Stool	April, 2022
KEMRI_2023Br102	*S*. Breda	582	32	Female	Stool	January, 2022
KEMRI_2023Br19^#^	*S*. Breda	582	28	Male	Blood	May, 2021
KEMRI_2023Br20	*S*. Breda	582	24	Female	Stool	June, 2021
KEMRI_2023Eb4	*S*. Eastbourne	414	12	Male	Stool	January, 2021
KEMRI_2023Eb52	*S*. Eastbourne	414	28	Male	Stool	May, 2022
KEMRI_2023On108	*S*. Orion	639	38	Male	Stool	June, 2021
KEMRI_2023On98	*S*. Orion	639	36	Male	Stool	April, 2022
KEMRI_2023Mt107	*S*. Muenster	321	27	Male	Stool	July, 2021
KEMRI_2023Gn109	*S*. Gaminara	239	18	Male	Stool	June, 2021
KEMRI_2023Hn49	*S*. Hann	11827	67	Female	Stool	April, 2022
KEMRI_2023Ab105	*S*. Aberdeen	4038	13	Male	Stool	November, 2021
KEMRI_2023Cg103	*S*. Chicago	937	31	Male	Stool	January, 2022
KEMRI_2023Mc51	*S*. Muenchen	82	24	Female	Stool	May, 2022
KEMRI_2023Ub110	*S*. Umbilo	2014	28	Male	Stool	May, 2021
KEMRI_2023Ky112	*S*. Kenya	991	17	Male	Stool	December, 2022
KEMRI_2023If118	*S*. Infantis	32	32	Female	Stool	May, 2023
KEMRI_2023Bc104	*S*. Banco	11832	31	Male	Stool	November, 2021
KEMRI_2023Kb17	*S*. Kiambu	309	26	Male	Stool	April, 2021
KEMRI_2023Kt40	*S*. Kentucky	314	24	Female	Stool	November, 2021

^*^Isolates carrying antimicrobial resistance genes.

^#^Isolate from blood sample.

**Table 2:** Predicted antigenic profiles and serogroups of non-typhoidal *Salmonella* serovars based on O Antigen, H1 (*fliC*), and H2 (*fljB*) predictions.

**Serovar**	**O antigen**	**H1 *fliC* prediction**	**H2 *fljB* prediction**	**Predicted antigenic profile**	**Serogroup**
*S*. Newport	8	e, h	1, 2	8: e, h:1,2	C2-C3
*S*. Breda	6, 8	z4, z23	e, n, z15	6, 8: z4, z23: e, n, z15	C2-C3
*S*. Eastbourne	9	e, h	1, 5	9: 2, h: 1, 5	D1
*S*. Orion	3, 10	y	1, 5	3, 10: y: 1, 5	E1
*S*. Kiambu	4, 12	z	1, 5	4, 12: z: 1, 5	B
*S*. Kentucky	8	i	z6	8: i: z6	C2-C3
*S*. Hann	40	k	e, n, z15	40: k: e, n, z15	R
*S*. Muenchen	8	d	1, 2	8: d: 1, 2	C2-C3
*S*. Chicago	28	r	1, 5	28: r: 1, 5	M
*S*. Banco	28	r	1, 7	28: r: 1,7	M
*S*. Aberdeen	11	i	1, 2	11: i: 1, 2	F
*S*. Muenster	3, 10	e, h	1, 5	3, 10: e, h: 1, 5	E1
*S*. Gaminara	16	d	1, 7	16: d: 1, 7	I
*S*. Umbilo	28	z10	e, n, x	28: z10: e, n, x	M
*S*. Kenya	7	l, z13	e, n, x	7: l, z13: e, n, x	C1
*S*. Infantis	7	r	1, 5	7: r: 1, 5	C1

### 3.3 Antimicrobial resistance

Resistance to the tested antimicrobials was observed in only 3 out of 25 *Salmonella* isolates. Two *S*. Newport strains, both classified as ST31, exhibited resistance to ampicillin and tetracycline. Additionally, one *S*. Newport strain (ST166) showed resistance to trimethoprim-sulfamethoxazole and tetracycline. Genomic analysis revealed that all three isolates carried acquired antimicrobial resistance (AMR) genes, but none exhibited known resistance-associated point mutations. The two *S*. Newport ST31 strains each carried the *bla*_*TEM*−1_ and *tet(A)* resistance genes. The *S*. Newport ST166 strain carried multiple resistance genes, including *tet(A), aph(6)-Id, dfrA14, aph(3*′'*)-Ib*, and *sul2*.

### 3.4 Plasmids carrying AMR genes

The two *S*. Newport ST31 isolates, KEMRI2023_Np50 and KEMRI2023_Np106, each carried two AMR genes on separate contigs. The beta-lactamase gene *bla*_*TEM*−1_ was located on a contig with sequences closely resembling those of *Shigella dysenteriae* strain BU53M1′s plasmid (GenBank accession: NZ_CP024467.1). The tetracycline resistance gene *tet(A)* was found on a contig that shared high sequence similarity with the plasmid p65COLEC-2 from *Escherichia coli* strain 65COLEC (GenBank accession: NZ_CP070916.1). Meanwhile, *S*. Newport ST166 (strain KEMRI2023_Np96) contained all of its AMR genes *(tet(A), aph(6)-Id, dfrA14, aph(3*′'*)-Ib, sul2*) on a single contig, which showed high sequence similarity to a multidrug-resistant (MDR) plasmid from *Citrobacter freundii* (plasmid CF_20.1, GenBank accession: NZ_MW115421.1) (Table 3).

**Table 3:** Contigs carrying AMR genes in three *S*. Newport strains and corresponding GenBank accession numbers of plasmids with similar sequences.

***Salmonella* strain**	**AMR genes**	**Contig containing AMR genes (GenBank Accession)**	**Contig length (bp)**	**GenBank accession number of plasmids with sequences similar to contigs carrying AMR genes**	**Inc type**	**Score**
KEMRI2023_Np50 (*S*. Newport ST31)	*bla_*TEM*−1_*	JBICQK010000012.1	46,914	NZ_CP024467.1	IncX1	0.99
	*tet(A)*	JBICQK010000022.1	4701	NZ_CP070916.1	IncH1	0.99
KEMRI2023_Np106 (*S*. Newport ST31)	*bla_*TEM*−1_*	JBICPW010000010.1	46,914	NZ_CP024467.1	IncX1	0.99
	*tet(A)*	JBICPW010000020.1	4,701	NZ_CP070916.1	IncH1	0.99
KEMRI2023_Np96 (*S*. Newport ST166)	*tet(A)*	JBICQG010000015.1	8,969	NZ_MW115421.1 (*Citrobacter freundii* strain CF_20.1 plasmid CF_20.1 MDR)	Col (pHAD28)	0.99
	*aph(6)-Id*					
	*dfrA14*					
	*aph(3′')-Ib*					
	*sul2*					

### 3.5 Biofilm formation

**Figure 3:**
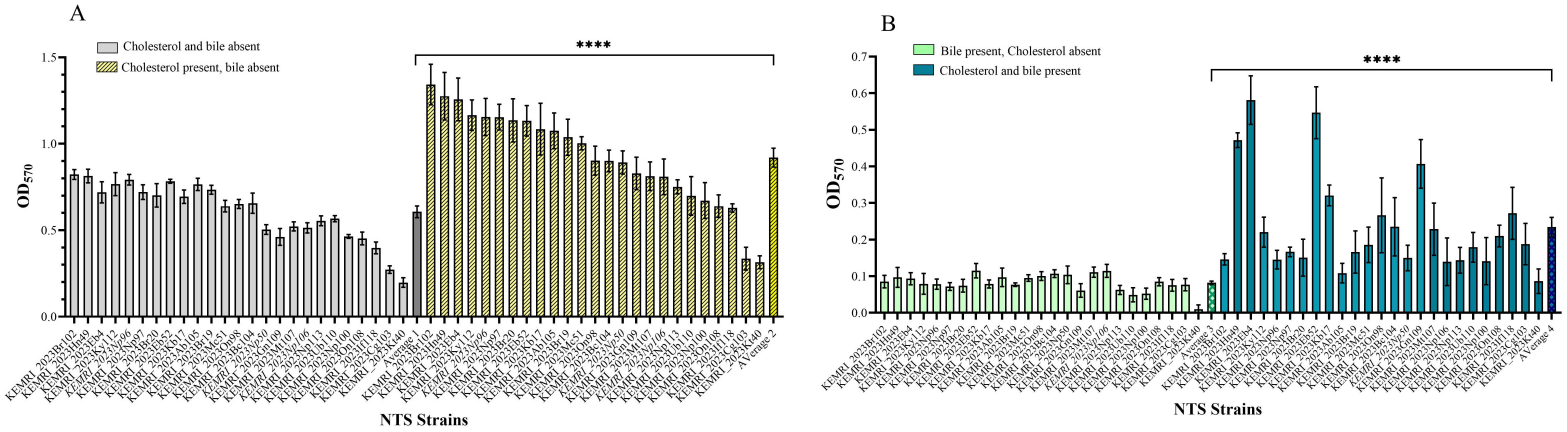
Biofilm formation by NTS serovars/strains under different conditions. **(A)** Biofilm quantification after growth on wells without cholesterol and bile and cholesterol coated wells but without bile, (unpaired *t* test 95% CI, 0.1840–0.4428). **(B)** Biofilm formation in media supplemented with 2.5% human bile (unpaired *t* test 95% CI, 0.09783–0.2066). For individual strains, error bars represent standard deviation, while averages are shown with error bars indicating the standard error of the mean (SEM). Statistical significance is denoted by **** for *p* < 0.001.

The ability of the NTS isolates to form biofilms under various *in vitro* conditions showed significant statistical variation. Biofilm formation differed significantly across four tested conditions: (i) in the absence of both cholesterol and bile, (ii) in the presence of cholesterol but absence of bile, Figure 3A, (iii) in the presence of bile but without cholesterol and (iv) in the presence of both cholesterol and bile (*p* < 0.0001) (Figure 3B). Notably, the isolates formed significantly stronger biofilms in cholesterol-coated wells but failed to form biofilms when exposed to 2.5% human bile. Among the 25 strains tested, 12 exhibited strong biofilm formation (OD_570_ > 1.0), 11 displayed moderate biofilm formation (OD_570_ between 0.5 and 1.0), and only 2 produced weak biofilms (OD_570_ < 0.5) on cholesterol-coated wells without bile. Serovars that formed strong biofilms included *S*. Breda (including strain KEMRI_2023Br19 isolated from blood sample), *S*. Hann, and *S*. Eastbourne, while *S*. Chicago and *S*. Kentucky were among those that formed weak biofilms.

## 4 Discussion

This study highlights the diversity of *Salmonella* serovars, excluding *S*. Typhimurium and *S*. Enteritidis, circulating in the Mukuru slums of Nairobi, Kenya. A total of 16 rarely reported NTS serovars were identified from samples collected from 25 patients, with *S*. Newport being the most prevalent. The genetic diversity within *S*. Newport, as evidenced by the presence of three sequence types (ST46, ST31, and ST166), suggests multiple transmission sources or evolutionary lineages. Other notable serovars included *S*. Breda, *S*. Eastbourne, and *S*. Orion, reflecting a broad spectrum of NTS serovars circulating in the population. In sSA, these serovars are sometimes reported as non-typable due to inconclusive identification using the Kauffmann-White classification system, largely attributed to the unavailability of specific monovalent antisera (Gilchrist and MacLennan, 2019). The NTS serovars have been implicated in NTS infections and previously accounted for 11.1% of non-typhoidal *Salmonella* infections in Kilifi, Kenya (Muthumbi et al., 2015), 12.4% in Mozambique (Mandomando et al., 2015), 0.9% in the Democratic Republic of Congo (Kalonji et al., 2015), 5.1% in Malawi (Feasey et al., 2015), and 17.9% in South Africa (Keddy et al., 2017).

Previously, we reported the prevalence and predominance of *S*. Typhimurium (ST19 and ST313) and *S*. Enteritidis (ST11) in the same study setting. These serovars were particularly common among school-aged children ( ≤ 16 years) and those under five, where they were associated with iNTS (Kariuki et al., 2019). While water, sanitation, and hygiene (WASH)-related infections, including typhoid fever, remain endemic in Mukuru (Kariuki et al., 2021; Muturi et al., 2024a), the detection of novel NTS serovars in this population suggests possible new introductions or emerging transmission pathways. Similarly, Feasey et al. (2012) and Park et al. (2021) highlighted the predominance of *S*. Typhimurium and *S*. Enteritidis in sSA, while noting an increasing detection of other serovars.

The positivity rate of these NTS serovars (0.9%) and the overlapping hotspots with typhoid fever (Muturi et al., 2024a), point to potential shared transmission pathways or risk factors. The predominance of stool-derived NTS isolates indicates that most of the serovars were primarily gastrointestinal.

Antimicrobial resistance genes were identified in 3 out of 25 isolates, all belonging to the serovar *S*. Newport. The resistance profiles varied among these isolates: ST31, carrying the *bla*_*TEM*−1_ and *tet(A)* genes, exhibited resistance to ampicillin and tetracycline; while ST166, harboring *bla*_*TEM*−1_, *tet(A), aph(6)-Id*, and *sul2* genes, showed resistance to sulfamethoxazole-trimethoprim (Co-trimoxazole) and tetracycline. Genomic analyses revealed that these AMR genes were associated with plasmids showing high sequence similarity to those from other members of the Enterobacteriaceae family, such as *Shigella dysenteriae, Escherichia coli* and *Citrobacter freundii* (Rasool et al., 2021; Schroeder et al., 2018). This finding suggests a potential role for horizontal gene transfer in the dissemination of AMR within the study setting. Antimicrobial resistance in NTS strains poses a significant public health challenge in sSA (Amir et al., 2024). Invasive NTS infections, a major cause of bacteremia, particularly in children and immunocompromised individuals, are increasingly characterized by multidrug resistance (Park et al., 2021). Reports of MDR strains, such as *S*. Typhimurium ST313 and *S*. Enteritidis ST11, have been documented across sSA, including in Kenya, Malawi, the Democratic Republic of Congo (DRC) and Ghana, complicating treatment efforts (Aldrich et al., 2019; Kariuki et al., 2022; Mahon and Fields, 2016). Additionally, the high prevalence of co-infections with diseases such as HIV and malaria further exacerbates the challenges in managing iNTS (Feasey et al., 2012).

The phylogenetic tree revealed the genetic diversity among the analyzed NTS isolates, with strains from this study clustering with those from previous studies, indicating shared evolutionary relationships. Based on genetic distances, the isolates exhibited varying degrees of relatedness to previously characterized strains. Notably, *S*. Newport ST31 strains (KEMRI_2023Np50 and KEMRI_2023Np106) carrying AMR genes clustered closely with *S*. Newport ST132 (GenBank accession: SAMN44048945). Similarly, the AMR-carrying *S*. Newport ST166 strain (KEMRI_2023Np96) showed high similarity to *S*. Newport ST166 (GenBank accession: SAMN37820486).

The ability of NTS isolates to form biofilms varied significantly under different *in vitro* conditions. Cholesterol enhanced biofilm formation, while bile inhibited it, suggesting that environmental factors and host-derived components play a critical role in biofilm dynamics as previously observed (Steenackers et al., 2012). Among the 25 strains tested, 12 exhibited strong biofilm formation *in vitro* (OD_570_ > 1.0), while 11 demonstrated moderate biofilm formation (OD_570_ between 0.5 and 1.0) in the presence of cholesterol. Bacteria in biofilms are usually enveloped in bacteria-initiated extracellular polymeric substances allowing the organisms to survive in hostile conditions, including exposure to Ultra Violet (UV) light, acid, dehydration, salinity, metal toxicity and several antibiotics (Hall-Stoodley et al., 2004; González et al., 2018). *In vivo* studies have shown that a cholesterol-rich diet promotes *Salmonella* spp. biofilm formation in the gastrointestinal tract by enhancing bacterial colonization, particularly in the cecum, and facilitating biofilm development on cholesterol-rich surfaces, leading to persistent infections (Cruz-Cruz et al., 2025). The strong biofilm-forming ability observed in 12 of the 25 *Salmonella* isolates may provide a protective advantage against commonly used antimicrobials for treating salmonellosis. These biofilms contribute to ~80% of chronic bacterial infections in humans, leading to increased hospitalization rates, elevated healthcare costs, and higher morbidity and mortality (Römling and Balsalobre, 2012). Further research is needed to identify the specific factors responsible for serovar-specific variations in biofilm-formation and to elucidate the regulatory genes involved across the 16 NTS serovars studied. Cholesterol's role in enhancing *Salmonella* biofilm-formation is well-documented (Gonzalez-Escobedo and Gunn, 2013; Muturi et al., 2024b). Bile, a complex digestive secretion composed of bile acids, bilirubin, phospholipids, and cholesterol, possess strong antimicrobial properties (An et al., 2022; Staley et al., 2017). Despite this, it has been shown to promote biofilm-formation in *S*. Typhimurium (Prouty et al., 2004) and *S*. Typhi (Muturi et al., 2024b). Although few studies have investigated the biofilm-forming ability of different NTS serovars and lineages under *in vivo* conditions, variations in biofilm formation and virulence among iNTS strains have been reported (Vasicek and Gunn, 2023). These findings highlight the complexity of biofilm-formation in NTS and suggest that serovar-specific factors and environmental conditions must be considered when studying biofilm formation and its role in pathogenesis.

This study is not without limitations, the most significant being the lack of non-human samples, which restricted the ability to trace potential sources of the NTS bacteria. Since all participants were outpatients, it is likely that they acquired the bacteria from community sources.

## 5 Conclusion

This study highlights the diversity of *Salmonella enterica* serovars, other than *S*. Typhimurium and *S*. Enteritidis, circulating in the Mukuru region of Nairobi, Kenya, along with the variability in AMR profiles and differences in biofilm formation among the serovars. The findings emphasize the importance of localized studies to understand regional variations in NTS epidemiology. To effectively mitigate the burden of NTS infections and curb the spread of AMR, sustained genomic surveillance, the development of advanced diagnostic tools for emerging *Salmonella enterica* infections, and the implementation of integrated public health interventions are essential.

## Data Availability

The datasets presented in this study can be found in online repositories. The names of the repository/repositories and accession number(s) can be found in the article/supplementary material.
